# Do Health and Demographic Surveillance Systems benefit local populations? Maternal care utilisation in Butajira HDSS, Ethiopia

**DOI:** 10.3402/gha.v7.24228

**Published:** 2014-07-04

**Authors:** Mesganaw Fantahun Afework, Seifu Hagos Gebregiorgis, Meselech Assegid Roro, Alemayehu Mekonnen Lemma, Saifuddin Ahmed

**Affiliations:** 1 Department of Reproductive Health and Health Service Management, School of Public Health, College of Health Sciences, Addis Ababa University, Addis Ababa, Ethiopia; 2 Department of Population, Family and Reproductive Health, Bill & Melinda Gates Institute for Population and Reproductive Health, Johns Hopkins Bloomberg School of Public Health, Johns Hopkins University, Baltimore, MD, USA

**Keywords:** demographic, surveillance, Ethiopia, antenatal care, skilled attendance, facility delivery

## Abstract

**Background:**

The benefits of Health and Demographic Surveillance sites for local populations have been the topic of discussion as countries such as Ethiopia take efforts to achieve their Millennium Development Goal targets, on which they lag behind. Ethiopia's maternal mortality ratio is very high, and in the 2011 Ethiopia Demographic and Health Survey (2011 EDHS) it was estimated to be 676/100,000 live births. Recent Global Burden of Disease (GBD) and estimates based on the United Nations model reported better, but still unacceptably high, figures of 497/100,000 and 420/100,000 live births for 2013. In the 2011 EDHS, antenatal care (ANC) utilization was estimated at 34%, and delivery in health facilities was only 10%.

**Objectives:**

To compare maternal health service utilization among populations in a Health and Demographic Surveillance System (HDSS) to non-HDSS populations in Butajira district, south central Ethiopia.

**Design:**

A community-based comparative cross-sectional study was conducted in January and February 2012 among women who had delivered in the 2 years before the survey.

**Results:**

A total of 2,296 women were included in the study. One thousand eight hundred and sixty two (81.1%) had attended ANC at least once, and 37% of the women had attended ANC at least four times. A quarter of the women delivered their last child in a health facility. Of the women living outside the HDSS areas, 715 (75.3%) attended ANC at least once compared to 85.1% of women living in the HDSS areas [adjusted odds ratio (AOR) 0.59; 95% CI 0.46, 0.74]. Of the women living outside the HDSS areas, only 170 (17.9%) delivered in health facilities and were assisted by skilled attendants during delivery, whereas 30.0% of those living in HDSS areas delivered in health facilities (AOR 0.66; 95% CI 0.48, 0.91).

**Conclusion:**

This paper provides possible evidence that living in an HDSS site has a positive influence on maternal health. In addition, there may be a positive influence on those living nearby or in the same district where an HDSS is located even when not included in the surveillance system.

Ethiopia's maternal mortality ratio (MMR) is very high and was estimated at 676/100,000 live births by the 2011 Ethiopia Demographic and Health Survey (2011 EDHS), while maternal health service utilization is low (1). Recent Global Burden of Disease (GBD) and estimates based on the United Nations model reported better, but unacceptably high, figures of 497/100,000 and 420/100,000 live births in 2013 (2, 3). In the 2011 EDHS, antenatal care (ANC) utilization was estimated at 34%, and only 10% of the deliveries took place in health facilities (1). Studies on maternal health are timely and relevant as the Millennium Development Goal (MDG) target year 2015 is approaching and Ethiopia and many sub-Saharan African countries are lagging behind their MDG targets (4, 5). Because of the difficulties in assessing MMR changes over short periods of time in developing countries where complete vital registration data are not available, maternal health service indicators are employed to measure maternal health progress toward achieving the MDG for maternal health (4).

Health and Demographic Surveillance System (HDSS) sites provide data on vital events and a sampling frame and base population for community-based research in countries where vital registration systems are non-existent or weak (6–9). An HDSS collects and monitors the demographic and health characteristics of a population living in a well-defined geographic area. The process starts with a baseline census followed by regular update of key demographic events (birth, death, and migration) and health events through systematic data collection procedures at set intervals (6).

There are many advantages of HDSS sites as a platform for research and research capacity building and in providing evidence-based interventions for health development (8–16). For example, it was reported that the Butajira HDSS site in Ethiopia was used in 20 PhD dissertations, 40 MPH/MSc theses, and over 100 articles that were published in reputable journals (*Unpublished proceedings of the School of Public Health, College of Health Sciences, Addis Ababa University Retreat, 2013*). It has also been speculated that HDSS sites may have better health indicators compared to populations not under surveillance because the repeated data collection and measurement could function as a passive intervention resulting in behavior change. In addition, populations from HDSS areas are often exposed to studies that may provide interventions (9). Comparability and representativity of HDSS populations in Ethiopia with the nation as a whole have been explored to a certain extent; mortality trends have been comparable (17), but the benefits of living in an HDSS site on health status have not been investigated.

The objective of this study is to compare maternal health service utilization in populations living in areas under HDSS and populations not under HDSS in Butajira district, south central Ethiopia.

## Methods

### Study design and period

We conducted a community-based comparative cross-sectional study in January and February 2012.

### Study area

The study was conducted in the Butajira district in south central Ethiopia. The district is located in the Southern Nations Nationalities and Peoples Region (SNNPR), which is one of nine administrative regions of Ethiopia. The district houses the Butajira Health and Demographic Surveillance System, also known as the Butajira Rural Health Program (BRHP), which was initiated in 1986. The BRHP includes one urban and nine rural communities (*kebeles*) that were randomly selected using the probability proportional to size (PPS) technique. The *kebele* is the smallest administrative unit, which consists of about 5,000 people. A baseline survey was conducted in 1986–87, which was followed by monthly visits to collect data on vital and related events (7). Additional censuses were conducted at 5-year intervals to check and validate surveillance data. After the 1999 census, a decision was made to conduct quarterly rounds of data collection instead of monthly visits in light of the experiences gained in Butajira and elsewhere (18).

### Study population and sampling

The study population includes all women who had delivered within 2 years of the data collection in the selected *kebeles* in the HDSS site and *kebeles* not in the DHSS site. Delivery in health facilities (skilled attendance at birth) was chosen as the variable of interest for this study because of its greater importance in predicting maternal health outcomes (4) and its low coverage (1). The skilled attendance at birth (SBA) is an indicator for MDG 5 (indicator 5.2) and we used delivery in a health facility as the measure SBA in this study. Very few or no skilled attendants are present during child birth at home in the study areas. Assuming a health facility delivery rate of 16% when the study was undertaken (19), 80% statistical power, a 95% confidence level, and an effect size of 6% difference between *kebeles* in the HDSS (HDSS *kebeles*) and *kebeles* not in the HDSS (non-HDSS *kebeles*), the calculated sample size for this study was 1,050 women who delivered in the previous 2 years. With a design effect of 1.6, 1,680 women who gave birth would be required. Thus, the number of women included in this study (2,296) provided adequate sample size and power. Six HDSS and six non-HDSS *kebeles* were selected for this study using simple random sampling among the *kebeles* in HDSS and non-HDSS *kebeles*, respectively.

### Data collection

Data collection was conducted by 10 trained and experienced high school graduate female interviewers and monitored by two supervisors. Enumeration of houses, household members, and women who had delivered during the previous 2 years was conducted first. Then, data were collected on socioeconomic and demographic characteristics and maternal health service utilization among women who delivered in the previous 2 years in both HDSS and non-HDSS *kebeles*. A pretest was conducted in a *kebele* outside Butajira district and the results were used to improve the study instrument.

### Data entry and analysis

Data were double entered by experienced data clerks. Data entry and analysis was performed using STATA 12. Frequency distribution of sociodemographic characteristics of the study population and the coverage of maternal health services were computed. A wealth index score was calculated for each household using the principal component analysis (PCA) method from the household durable goods and household structural conditions (e.g. materials used to construct walls, roof, floors of houses, type of toilet, and land possession). These variables have been used to categorize wealth in the EDHS (1). Households were ranked according to the total wealth score and then divided into wealth quintiles as a proxy of household economic status.

Multivariate logistic regression models were used to estimate odds ratios (95% confidence intervals) to determine the association between living in HDSS *kebeles* or non-HDSS *kebeles* and use of ANC and delivery in health facilities. Logistic regression analysis was employed to control potential confounding factors including place of residence, educational status, religion, number of deliveries, and wealth status.

## Results

The sociodemographic characteristics of the study population are shown in Table 1. A total of 2,296 women were included in the study. One thousand three hundred and forty-seven (58.7%) were from HDSS *kebeles*, while 949 (41.3%) were from non-HDSS *kebeles*. The majority (74.9%) were rural residents and belonged to the age group 20–29 (56.3%). One thousand three hundred and twenty-five (57.7%) were unable to read and write. About 97% of the women were married and 62% were Muslim. Occupation-wise, 62% were housewives and 4.4% combined household chores with farm work.

**Table 1:** Sociodemographic characteristics of women who delivered a baby in the 2 years preceding the survey in Butajira HDSS and non-HDSS sites, south central Ethiopia 2012 (N=2,296)

Characteristics	Number	Percent
HDSS site		
Yes	1,347	58.7
No	949	41.3
Age group		
15–19	116	5.1
20–29	1,290	56.3
30–39	772	33.1
40–49	112	4.9
Place of residence		
Urban	577	25.1
Rural	1,719	74.9
Level of education		
None (unable to read and write)	1,325	57.7
Primary	749	32.6
Secondary	165	7.2
College	57	2.5
Marital status		
Currently married	2,216	96.5
Widowed, divorced, never married	80	3.5
Religion		
Orthodox christian	681	29.7
Muslim	1,411	61.5
Protestant	192	8.4
Catholic	10	0.4
Occupation		
Farmer and housewife	100	4.4
Housewife	1,521	66.2
Employee	459	20.0
Others	216	9.4

### ANC attendance and delivery in a health facility

One thousand eight hundred and sixty-two (81.1%) women in the study had attended ANC at least once, and 37% of the women had attended ANC at least four times. Twenty-five percent of the women delivered their last child in a health facility.

### Association between living in an HDSS kebele and other factors with attending ANC at least once

Table 2 shows data regarding whether living in an HDSS *kebele* along with certain sociodemographic characteristics are associated with attending ANC at least once. Seven hundred and fifteen (75.3%) of the women living outside the HDSS areas attended ANC at least once compared to 85.1% of women living in the HDSS areas [adjusted odds ratio (AOR) 0.59 (95% CI 0.46, 0.74)]. When adjusted for other factors, wealth quintile and number of deliveries were statistically significantly associated with attending ANC at least once. The odds of attending ANC at least once was about 3.5 times higher among the richest compared to the poorest. Those who had delivered seven or more times had an approximately 40% lower chance of attending ANC at least once compared to those who had delivered once or twice. Age group, place of residence (urban vs. rural), religion, occupational status, educational status, and marital status did not show statistically significant association with ANC attendance at least once in this study population.

**Table 2:** Association between living in an HDSS site or not and sociodemographic characteristics with antenatal care attendance at least once, in Butajira district, south central Ethiopia 2012

	Had antenatal care			
			
Characteristics	Yes	No	Crude odds ratio (95% CI)	Adjusted odds ratio (95% CI)
HDSS site				
Yes	1,146 (85.1)	201 (14.9)	1.00	1.00
No	715 (75.3)	234 (24.7)	0.54 (0.43, 0.67)	0.59 (0.46, 0.74)*
Place of residence				
Urban	530 (91.8)	47 (8.2)	1.00	1.00
Rural	1,331 (77.4)	388 (22.6)	0.30 (0.22, 0.42)	0.84 (0.54, 1.30)
Age group (years)				
15–19	100 (86.2)	16 (13.8)	1.00	1.00
20–29	1,078 (83.6)	212 (16.4)	0.81 (0.45, 1.44)	1.07 (0.59, 1.92)
30–39	602 (78.0)	170 (22.0)	0.57 (0.31, 1.01)	1.06 (0.55, 2.03)
40–49	78 (69.6)	34 (30.4)	0.37 (0.18, 0.75)	0.83 (0.38, 1.81)
Women's educational status				
None	1,015 (76.6)	310 (23.4)	1.00	1.00
Primary	665 (84.8)	114 (15.2)	0.99 (0.72, 1.34)	1.18 (0.90, 1.54)
High school	156 (94.6)	9 (5.5)	5.29 (2.59, 11.22)	1.81 (0.84, 3.90)
College/University	55 (96.5)	2 (3.5)	8.40 (2.00, 50.05	2.16 (0.49, 9.50)
Wealth quintile				
Poorest	346 (75.2)	114 (24.8)	1.00	1.00
Poor	344 (74.9)	115 (25.1)	0.99 (0.72, 1.34)	0.96 (0.70, 1.30)
Middle	355 (77.3)	104 (22.7)	1.12 (0.82, 1.54)	1.13 (0.82, 1.56)
Rich	380 (82.8)	79 (17.2)	1.28 (0.92, 1.80)	1.31 (0.91, 1.91)
Richest	436 (95.0)	23 (5.0)	6.25 (3.82, 10.28)	3.56 (1.97, 6.41)*
Marital status				
Currently married	1,798 (81.1)	418 (18.9)	1.00	1.00
Currently unmarried	63 (78.8)	17 (21.3)	0.86 (0.49, 1.55)	0.69 (0.39, 1.24)
Occupation				
Farmer and housewife	70 (70.0)	30 (30.0)	1.00	1.00
House wife	1,227 (80.7)	294 (19.3)	1.79 (1.12, 2.85)	1.39 (0.87, 2.21)
Employee	387 (84.3)	72 (15.7)	2.30 (1.36, 3.89)	1.15 (0.68, 1.96)
Other	177 (81.9)	39 (18.1)	1.95 (1.08, 3.50)	1.40 (0.78, 2.51)
Religion				
Orthodox Christian	569 (83.6)	112 (16.4)	1.00	1.00
Muslim	1,119 (79.3)	292 (20.7)	0.75 (0.59, 0.97)	0.81 (0.62, 1.05)
Protestant	162 (84.4)	30 (15.6)	1.06 (0.67, 1.69)	1.03 (0.65, 1.63)
Catholic	9 (90.0)	1 (10.0)	1.77 (0.23, 37.70)	2.24 (0.28, 18.16)
Number of deliveries				
1–2	708 (87.5)	101 (12.5)	1.00	1.00
3–4	534 (79.7)	136 (20.3)	0.56 (0.42, 0.75)	0.76 (0.55, 1.04)
5–6	361 (77.8)	103 (22.2)	0.50 (0.37, 0.68)	0.73 (0.49, 1.09)
7+	258 (73.1)	95 (26.9)	0.39 (0.28, 0.54)	0.62 (0.39, 0.98)*

* Significant associations (P<0.05).

### Association between living in an HDSS kebele and other factors with ANC attendance at least four times

Five hundred and twenty-five (39.0%) of the women who lived in HDSS *kebeles* had attended ANC at least four times, and 316 (33.3%) of those who lived in non-HDSS areas had attended ANC at least four times. Living in HDSS *kebeles* did not have a significant association with ANC attendance at least four times [AOR 0.97 (95% CI: 0.78, 1.19)]. Variables that were significantly associated with ANC attendance at least four times included place of residence, wealth quintile, and number of deliveries. The odds of rural residents attending ANC was about 30% lower than those living in urban areas [AOR: 0.70 (95% CI: 0.51, 0.95)]. Those who belonged to the rich and richest quintiles were more likely to attend ANC at least four times compared to the poorest [AOR: 2.33 (95% CI: 1.67, 3.24)], [AOR: 3.91 (95% CI: 2.62, 5.84)], respectively. The odds of attending ANC at least four times by women who delivered 7 times or more was 42% lower than women had delivered once or twice [AOR: 0.58 (95% CI 0.38, 0.88)].

### Association between living in an HDSS kebele and other factors with delivery in health facility

As shown in Table 3, the odds of delivering in health facilities for women living in a non-HDSS *kebele* were lower than those for women living in an HDSS *kebele* [AOR: 0.66 (95% CI 0.48, 0.91)]. Strong statistically significant associations were found between women delivering in health facilities and their educational status, wealth status, and number of deliveries. Those who had a college education had a higher chance of delivering in health facilities [AOR: 4.84 (95% CI 1.98, 11.84)], while the odds of delivering in health facilities were much higher for the richest compared to the poorest women [AOR: 17.5 (95% CI: 9.85, 31.26)]. Number of lifetime deliveries (per woman) was inversely related to recently delivering in a health facility. The odds of a woman delivering in a health facility among those who had seven or more deliveries was less than a quarter of those who had one or two deliveries [AOR: 0.24 (95% CI: 0.14, 0.44)].

**Table 3:** Association of living in HDSS site or not and sociodemographic characteristics with place of delivery in Butajira district, south central Ethiopia 2012

	Place of delivery			
			
Characteristics	Health facility	Home	COR (95% CI)	AOR (95% CI)
HDSS site				
Yes	404 (30.0)	943 (70.0)	1.00	1.00
No	170 (17.9)	779 (82.1)	0.51 (0.41, 0.63)	0.66 (0.48, 0.91)*
Place of residence				
Urban	345 (59.8)	232 (40.2)	1.00	1.00
Rural	229 (13.3)	1,490 (86.7)	0.10 (0.08, 0.13)	0.70 (0.48, 1.03)
Age group (years)				
15–19	80 (35.7)	144 (64.3)	1.00	1.00
20–29	714 (29.3)	1,813 (71.7)	0.71 (0.53, 0.95)	0.77 (0.46, 1.25)
30–39	408 (21.5)	1,490 (78.5)	0.49 (0.36, 0.67)	1.13 (0.61, 2.09)
40–49	32 (11.0)	259 (89.0)	0.22 (0.13, 0.35)	1.85 (0.77, 4.49)
Women's educational status				
None	440 (14.2)	2,658 (85.8)	1.00	1.00
Primary	434 (31.8)	931 (68.2)	2.82 (2.41, 3.29)	1.20 (0.91, 1.60)
High school	291 (72.4)	111 (27.6)	15.84 (12.36, 20.39)	3.73 (2.27, 6.14)*
College/University	72 (85.7)	12 (14.3)	36.25 (18.93)	4.84 (1.98, 11.84)*
Wealth quintile				
Poorest	22 (4.8)	438 (95.2)	1.00	1.00
Poor	29 (6.3)	430 (93.7)	1.34 (0.73, 2.46)	1.17 (0.65, 2.09)
Middle	48 (10.5)	411 (89.5)	2.33 (1.34, 4.05)	1.97 (1.15, 3.81)*
Rich	148 (32.2)	311 (67.8)	9.47 (5.79, 15.62)	5.81 (3.44, 9.81)*
Richest	327 (71.2)	132 (28,8)	53.36 (32.43, 88.56)	17.5 (9.85, 31.26)*
Marital status				
Currently married	1,144 (24.3)	3,562 (75.7)	1.00	1.00
Currently unmarried	93 (38.3)	150 (61.7)	1.93 (1.46, 2.54)	1.49 (0.85, 2.63)
Religion				
Orthodox Christian	205 (30.1)	476 (69.9)	1.00	1.00
Muslim	315 (22.3)	1,096 (77.7)	0.67 (0.54, 0.82)	0.82 (0.62, 1.08)
Protestant	52 (27.1)	140 (72.9)	0.86 (0.59, 1.25)	0.87 (0.55, 1.39)
Catholic	1 (10)	9 (90)	0.26 (0.01, 2.00)	1.07 (0.12, 9.58)
Occupation				
Farmer and house wife	5 (5.0)	95 (95.0)	1.00	1.00
Housewife	331 (21.8)	1,190 (78.2)	5.28 (2.05, 14.85)	1.95 (0.74, 5.19)
Employee	188 (41.0)	271 (59.0)	13.18 (5.05, 37.48)	1.95 (0.71, 5.36)
Other	50 (23.2)	166 (76.8)	5.72 (2.10, 16.92)	2.03 (0.71, 5.74)
Number of deliveries				
1–2	722 (40.7)	1,053 (59.3)	1.00	1.00
3–4	282 (20.1)	1,119 (80.0)	0.37 (0.31, 0.43)	0.43 (0.31, 0.60)*
5–6	158 (15.4)	868 (84.6)	0.27 (0.22, 0.32)	0.39 (0.25, 0.61)*
7+	73 (9.8)	669 (90.2)	0.15 (0.12, 0.20)	0.24 (0.14, 0.44)*

* Significant associations (P<0.05).

## Discussion

We used a community-based study to assess whether living in an area (*kebele*) in which an HDSS is being run contributes to better maternal health service utilization or not.

The results of this study indicate that a woman who lives in a non-HDSS *kebele* is less likely to use ANC at least once compared to a woman living in an HDSS *kebele*. This difference might be related to the better awareness about maternal health care that the population of HDSS sites has due to exposure to several years of surveillance and research activities.

The WHO advocates a minimum of four target-oriented ANC visits during pregnancy to deal with problems that may arise at different periods of pregnancy and to improve pregnancy outcomes (20). Although a higher proportion of women in HDSS *kebeles* had attended ANC at least four times, the difference between non-HDSS and HDSS *kebeles* was not statistically significant when adjusted for other factors. Women in general may find it difficult to repeatedly go to health facilities during pregnancy even if there is better awareness about the advantages of ANC among women living in HDSS *kebeles*.

The odds of women delivering their babies in a health facility in non-HDSS *kebeles* are about half of those women living in HDSS *kebeles*, indicating a clear advantage for women living in HDSS *kebeles*. Health facility delivery (skilled attendance at birth) is considered one of the most important, if not the most important, predictor of maternal mortality (4). This is because maternal mortality and complications are not predictable and most maternal deaths occur around the time of delivery. Thus, living in HDSS *kebeles* is likely to be associated with lower maternal mortality than in non-HDSS *kebeles*.

Overall, ANC attendance and health facility delivery for women living in the study areas appear much higher than the national and regional averages reported in the 2011 EDHS (1). The national ANC coverage (where coverage is at least one visit) was reported to be 34%, and coverage in the region where BRHP is located, the SNNPR, was 27%. Delivery in health facilities was reported to be about 10% in the region, whereas the results of this study indicate ANC coverage of 80% and health facility delivery of 25%. The EDHS results are presented as averages for the nation as a whole or for administrative regions such as SNNPR. Therefore, it is difficult to compare the results of the study for this district with that of the EDHS reports. In addition, the data for EDHS coverage pertains to 5 years preceding the survey (i.e. 2011), whereas this study deals with women who delivered in the 2 years prior to mid-2012. Assuming that the average EDHS results for the country or region represent the study district, a possible explanation for the current health service utilization is that there may have been a general increase after the results of the EDHS survey were announced and more vigorous work was done in the country to improve maternal health service utilization as achieving MDG 5 became worrisome. However, it can be argued that such a change might not have been achieved in such a short time. Thus, the HDSS *kebeles*, and to a lesser but appreciable extent the neighboring non-HDSS *kebeles*, may have benefited from activities in the HDSS sites.

It has been reported that studies conducted within the Butajira HDSS site accrued some health benefits including the treatment of certain childhood diseases under study in the past (7, 18). However, specific interventions to address maternal health or maternal health service utilization have not been documented in the past 15 years. Thus, the results of this study indicate that improved maternal health service utilization can probably be attributed to the general effect of the ongoing surveillance activities.

Benefits for local populations residing in HDSS sites such as Butajira have often been questioned by community members, health authorities, visitors, and researchers; this question provided the motivation for conducting the current study (21). BRHP has made attempts to provide data on vital events and results of studies to local health authorities and administrators in annual workshops and bulletins for use in health planning and decision making, although this has not been done regularly and consistently during recent times. However, certain PhD theses works in Butajira have challenged the issue of data ownership and use by the community and concerned government sectors (21). It was emphasized that the most immediate and, in hindsight, the most obvious knowledge from the 21 years of BRHP had not been systematically reported where it belonged – in the local community of the Butajira District – despite continuous collection of relevant data. Fatigue of the community and lack of immediate benefits were considered to be challenges for continuous data collection for INDEPTH sites that include the Butajira HDSS site (22).

In conclusion, this paper provides likely evidence of the positive influence of living in an HDSS site for maternal health and perhaps of the positive influence of residing in the same district where an HDSS is located, even when not included in the system. Periodic, well-designed research will still be necessary in order to produce data on the benefits of HDSS for local populations. This is particularly important in countries such as Ethiopia where a number of HDSS sites have been recently established. The need to give due attention to the local benefits of living in HDSS sites through proper planning, implementation, and monitoring and evaluation of activities in established and emerging HDSS sites cannot be undermined.

We recommend that further studies explore the concrete interventions in HDSS sites that make a difference in health service utilization and other outcomes.
